# The Silent Culprit: Paroxysmal Nocturnal Hemoglobinuria Masquerading as Cryptogenic Stroke

**DOI:** 10.7759/cureus.84930

**Published:** 2025-05-27

**Authors:** Aaron S Sidhu, Harneet Grewal, Joey Bettencourt, Riya Sharma

**Affiliations:** 1 Internal Medicine, Abrazo Community Health Network, Glendale, USA; 2 Internal Medicine, Dayanand Medical College and Hospital, Ludhiana, IND

**Keywords:** arterial thromboembolism, complement deficiency, conventional cerebral angiogram, flow cytometry, human monoclonal antibody, malignant hematology, mechanical thromb, paroxysmal nocturnal hemoglobinuria (pnh), stroke complications, transesophageal echocardiogram (tee)

## Abstract

We present a case of a previously healthy 20-year-old male patient who experienced recurrent arterial thromboembolic events without identifiable risk factors. Extensive evaluation ultimately revealed paroxysmal nocturnal hemoglobinuria (PNH), a rare clonal hematopoietic disorder. While PNH is classically associated with venous thrombosis, arterial thromboembolic events are uncommon and may obscure the diagnosis, especially in young patients without underlying vascular risk factors.

Clinicians should maintain awareness of PNH’s variable presentation and its potential to masquerade as more common thrombotic or autoimmune conditions. Early recognition and appropriate therapy can prevent further complications and improve patient outcomes. This case contributes to the growing recognition of arterial events as a possible manifestation of PNH and reinforces the value of comprehensive diagnostic evaluation in cases of recurrent stroke without an obvious cause.

## Introduction

Paroxysmal nocturnal hemoglobinuria (PNH) is a rare, acquired clonal hematopoietic stem cell disorder characterized by complement-mediated intravascular hemolysis, thrombosis, bone marrow failure, and increased susceptibility to infections. It affects approximately one to two individuals per million annually, with a prevalence of 10 to 20 per million [1]. PNH can present at any age, with a median age in the early to mid-30s, and occurs equally in both male and female patients. Despite advancements in supportive care, the five- and 10-year mortality rates remain high, at approximately 35% and 50%, respectively [1].

The underlying pathophysiology involves a deficiency of the complement regulatory proteins CD55 and CD59 on the surface of red blood cells, granulocytes, and platelets, leading to uncontrolled complement activation and cell lysis [2-4]. The introduction of eculizumab, a monoclonal antibody targeting complement protein C5, has significantly improved outcomes by inhibiting the terminal complement cascade and reducing both hemolysis and thrombotic risk [5].

Thrombosis remains the leading cause of morbidity and mortality in PNH, accounting for 40%-67% of deaths [6]. While thromboses most often occur in the venous system-such as in deep veins, pulmonary arteries, and hepatic veins-arterial events and thrombosis at atypical sites, including cerebral and mesenteric veins, have been reported [7]. Thrombotic risk correlates with PNH clone size; patients with ≥50% PNH granulocytes have a significantly higher risk compared to those with smaller clones [8].

Cerebrovascular complications, including stroke, though less common, are increasingly recognized in PNH. Most cases involve cerebral venous thrombosis, but arterial ischemic strokes have also been described. These events highlight the diagnostic challenges and clinical vigilance required when evaluating neurologic symptoms in patients with PNH.

We present a case of recurrent cerebral infarctions in a patient with PNH, initially attributed to arterial thrombosis. This case underscores the importance of clinicians remaining vigilant in suspecting PNH when encountering young patients with cryptogenic arterial thrombosis, as prompt diagnosis and treatment can significantly improve patient outcomes.

This article was previously presented as a meeting abstract at the 2023 CHEST Annual Meeting on October 11, 2023.

## Case presentation

A 22-year-old previously healthy male patient presented to the emergency department with acute-onset right-sided hemiparesis and dysarthria. He was hemodynamically stable and afebrile and had no prior personal or family history of thrombotic events, autoimmune disease, or known hematologic disorders.

**Figure 1:**
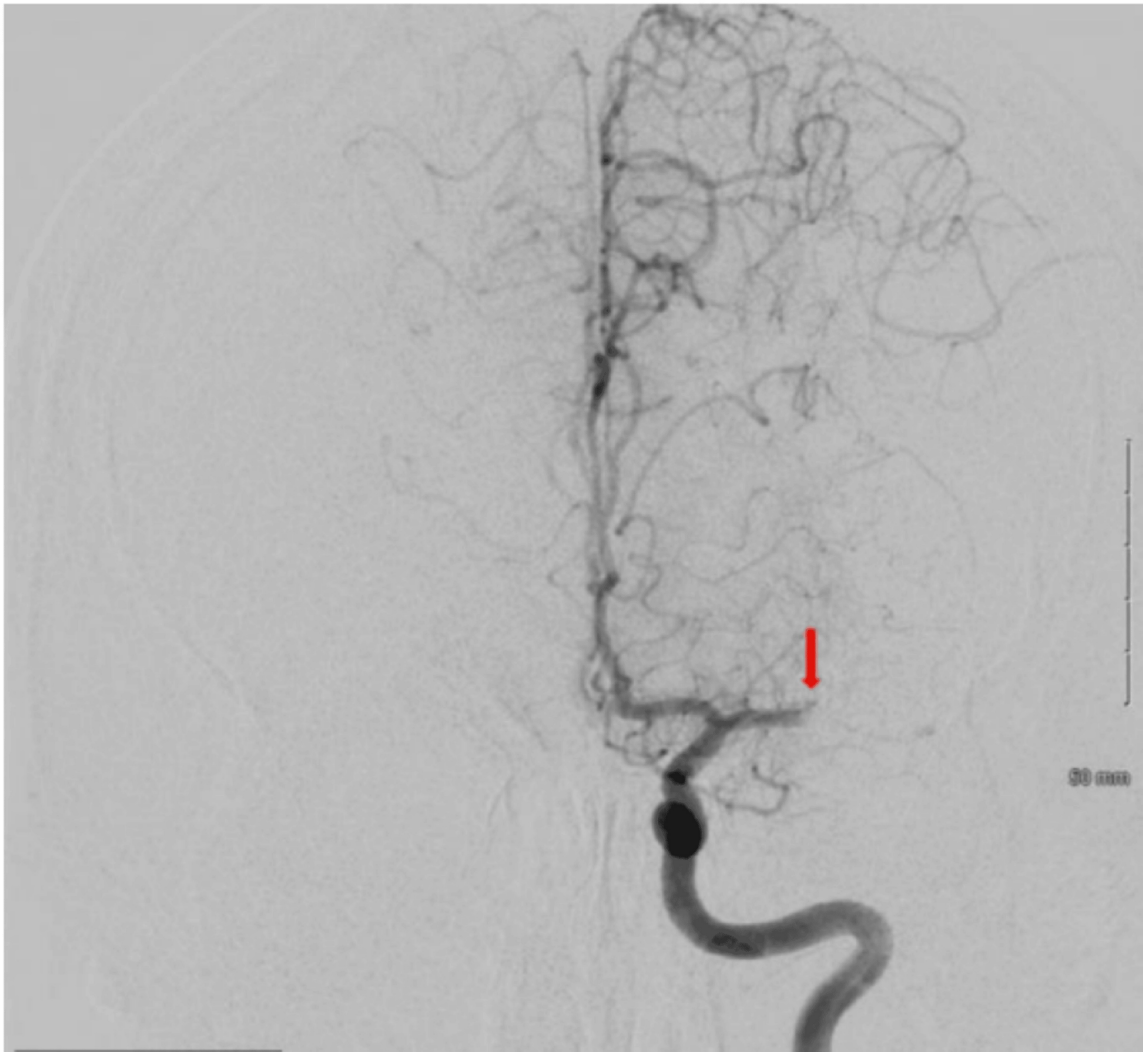
Cerebral angiogram demonstrating left MCA branch M1 segment occlusion MCA: middle cerebral artery

**Figure 2:**
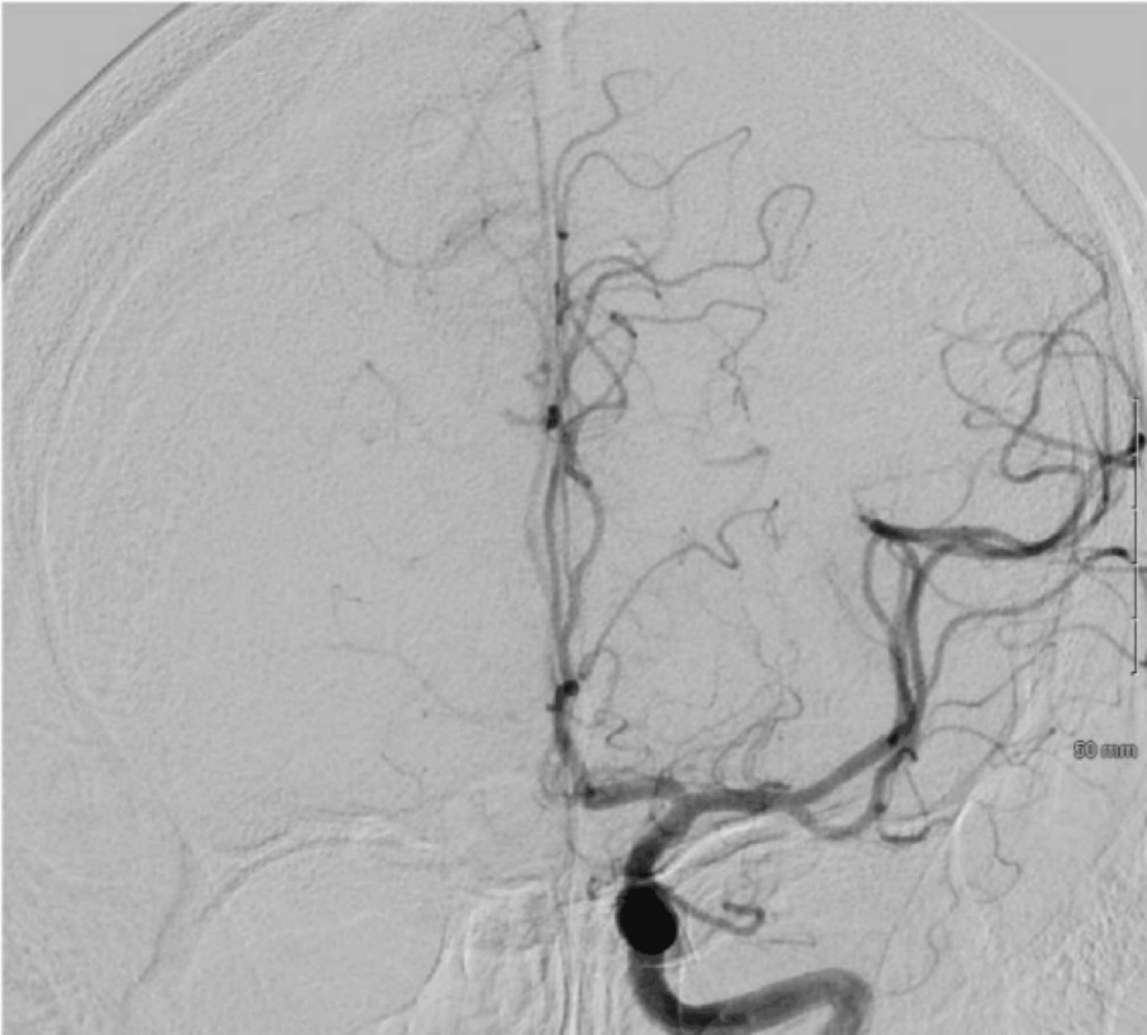
Status post mechanical thrombectomy achieving TICI 3 (complete reperfusion) TICI: thrombolysis in cerebral infarction

A computed tomography angiography (CTA) of the head and neck revealed an occlusion of the left middle cerebral artery (MCA). Given the acute ischemic presentation and time of symptom onset, the patient was administered intravenous tissue plasminogen activator (tPA) per stroke protocol. This was followed by a cerebral angiogram with an emergent mechanical thrombectomy performed by an interventional neurologist, achieving successful recanalization (Figures 1, 2).

Subsequent magnetic resonance imaging (MRI) of the brain revealed multiple infarcts within the left MCA territory and three additional acute infarcts in the right cerebral cortex. These bilateral findings were unusual and raised suspicion for a cardioembolic etiology. A transthoracic echocardiogram (TTE) with a bubble study demonstrated normal cardiac function and no evidence of intracardiac shunting, such as a patent foramen ovale or atrial septal defect. Bilateral lower extremity venous Doppler ultrasounds were also negative for deep vein thrombosis. Despite these unremarkable studies, a transesophageal echocardiogram (TEE) was pursued to further evaluate for a potential embolic source such as valvular vegetations or cardiac thrombus. The TEE was negative for intracardiac thrombus or structural abnormalities.

**Figure 3:**
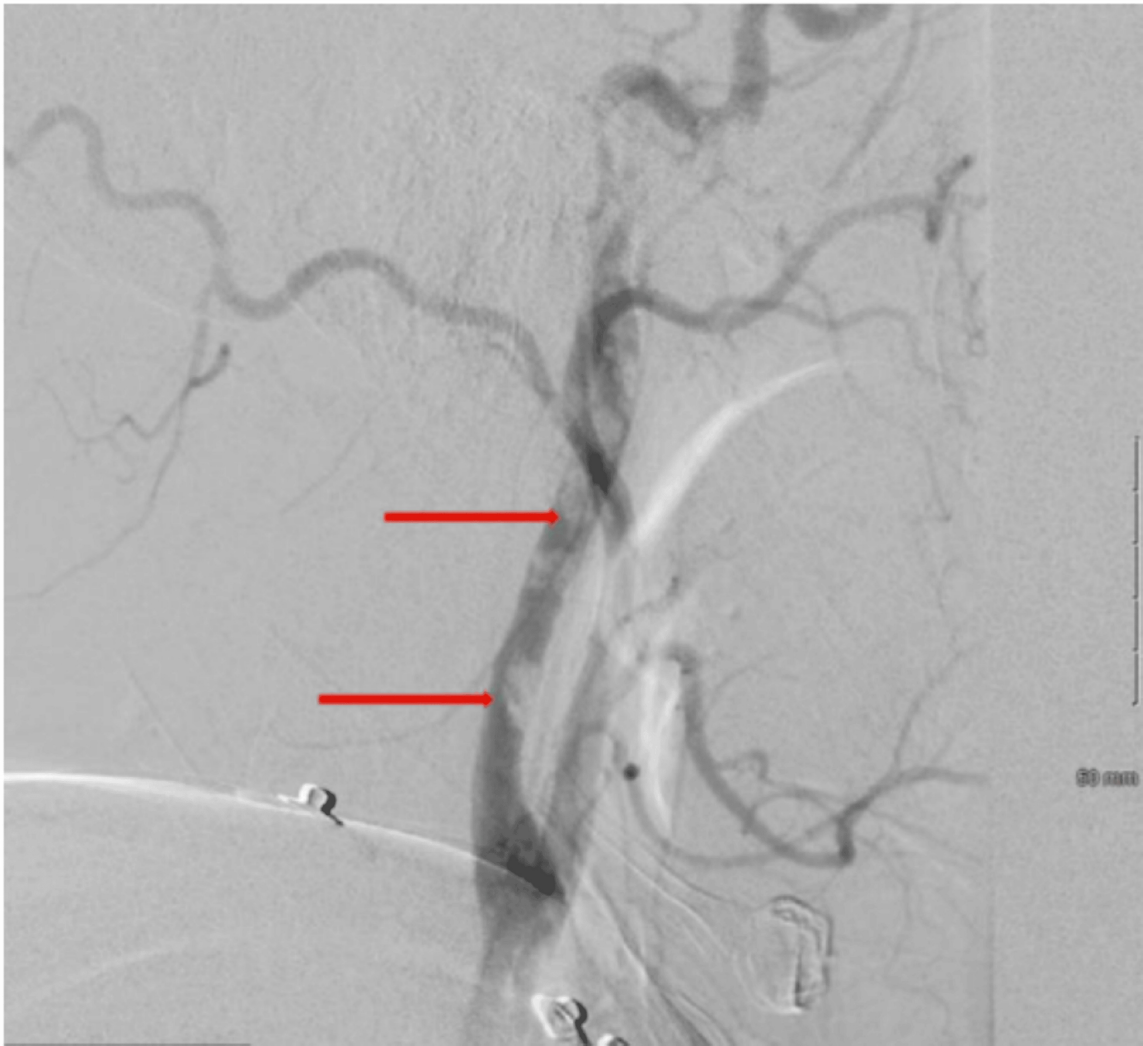
Cerebral angiogram from the second mechanical thrombectomy revealed a substantial clot burden in the left common carotid artery

**Figure 4:**
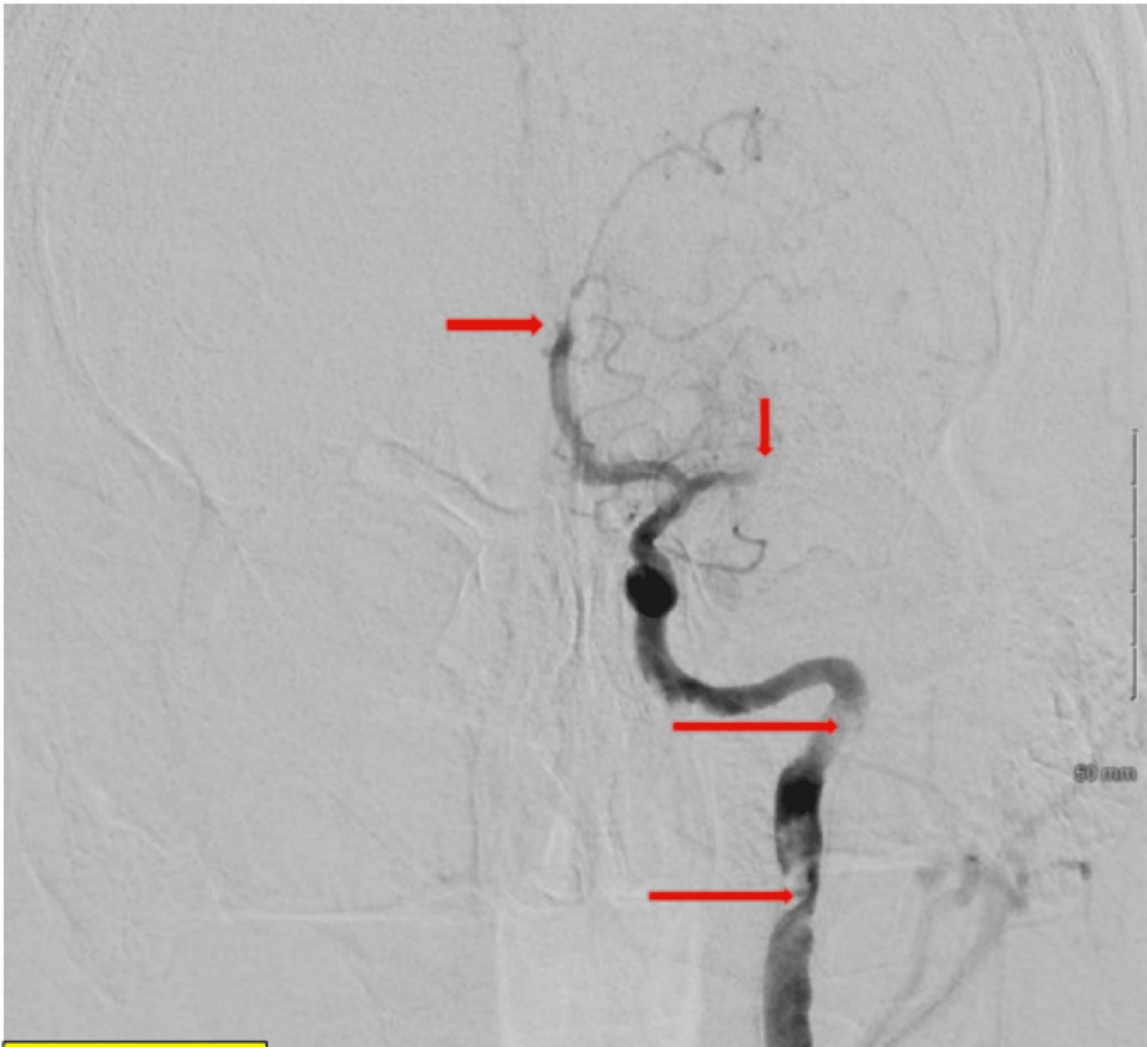
Cerebral angiogram from the second mechanical thrombectomy revealed a substantial clot burden in the left internal carotid arteries, mainly in the left cervical internal carotid artery, causing partial occlusion

**Figure 5:**
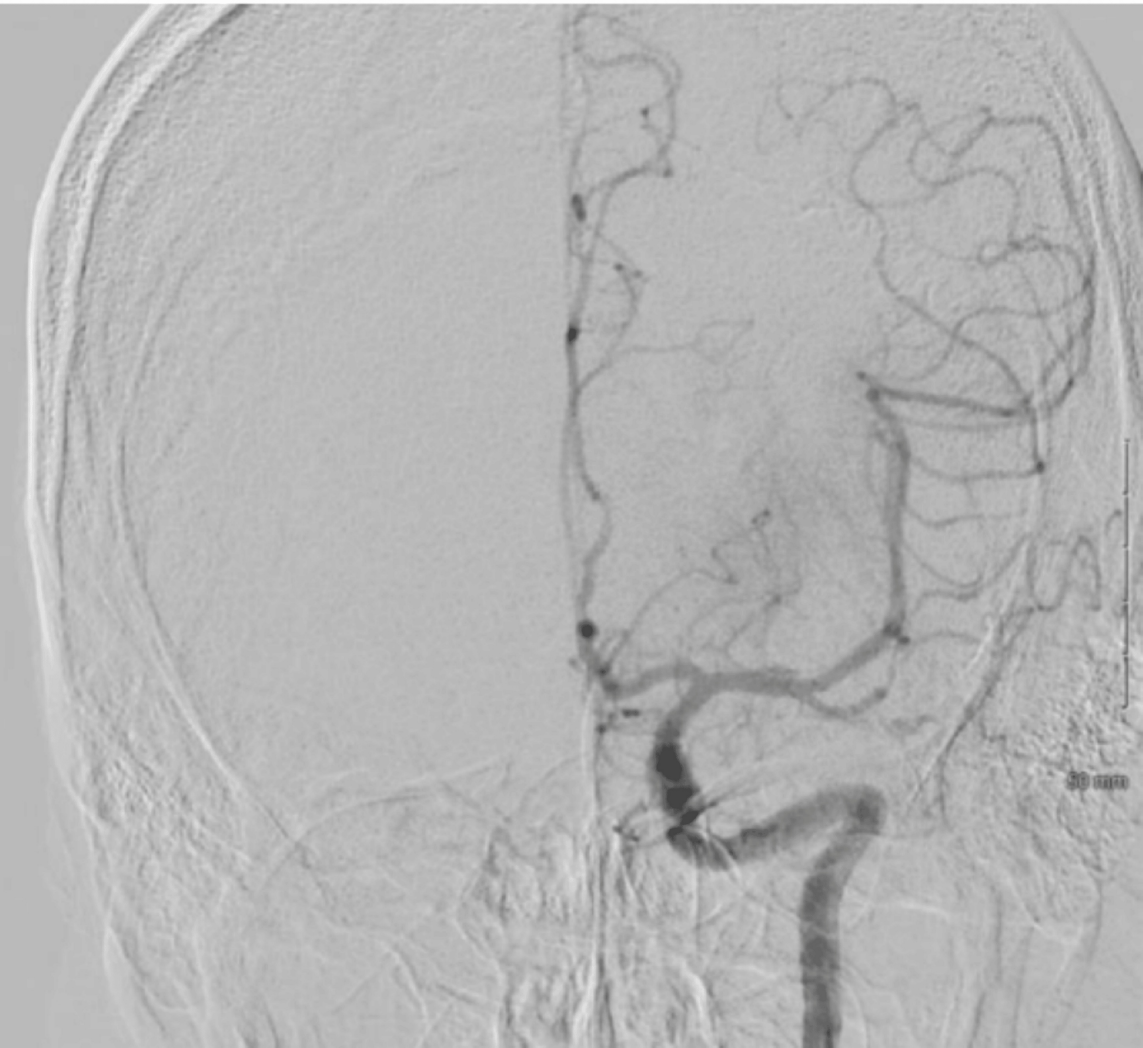
Second mechanical thrombectomy of left cervical internal carotid artery achieving TICI 2C recanalization (near complete reperfusion) TICI: thrombolysis in cerebral infarction

**Figure 6:**
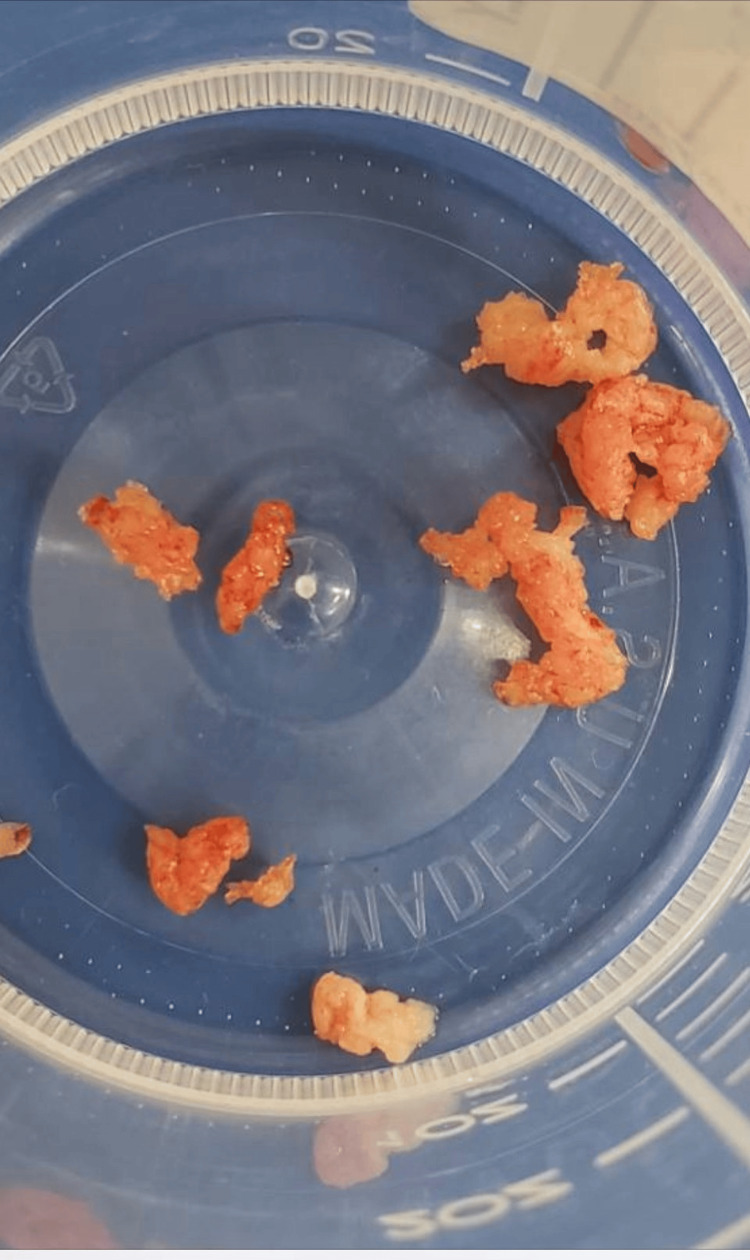
The retrieved clot demonstrated an unusual texture and color, contrasting with the typical dark and rubbery appearance. The clot’s fragile nature, indicative of a recent formation, prompted its submission for pathological analysis, resulting in its identification as a clot

Within 24 hours following the TEE, the patient experienced a recurrence of right-sided neurological deficits. Repeat imaging revealed new occlusions of the left anterior cerebral artery (ACA) and recurrent MCA involvement. CTA further identified multiple thrombi along the cervical segment of the left internal carotid artery (ICA) (Figures 3, 4). He underwent a second emergent mechanical thrombectomy with successful removal of the arterial thrombi (Figure 5). The retrieved clot demonstrated an unusual color and texture, prompting submission for further analysis (Figure 6).

Given the unusual pattern of recurrent, bilateral arterial thrombi in a young patient with no traditional cardiovascular risk factors, the hematology/oncology service was consulted. A comprehensive hypercoagulable workup was initiated, including testing for antiphospholipid syndrome, factor V Leiden mutation, prothrombin gene mutation, protein C and S deficiency, and antithrombin III deficiency-all of which returned within normal limits.

Routine laboratory evaluation revealed normocytic anemia (hemoglobin 9.8 g/dL), elevated lactate dehydrogenase (LDH), and undetectable haptoglobin, suggesting intravascular hemolysis. There was no evidence of schistocytosis on peripheral smear, and a direct antiglobulin (Coombs) test was negative, lowering the likelihood of autoimmune hemolytic anemia. Given these findings, flow cytometry was performed to evaluate for PNH. The assay revealed complete CD59 deficiency in 74% of erythrocytes and a similarly expanded PNH granulocyte clone, consistent with a definitive diagnosis of classical hemolytic PNH [9].

The patient was initiated on eculizumab, a terminal complement inhibitor, following administration of the meningococcal vaccine and a two-week course of prophylactic antibiotics to mitigate infection risk. He tolerated the therapy well and was discharged with close outpatient follow-up under a hematology/oncology specialist. Weekly eculizumab infusions were continued in the outpatient setting.

At his one-month follow-up, the patient showed significant neurological improvement with only mild residual expressive aphasia. Hematologic markers had stabilized, and no further thrombotic events had occurred.

## Discussion

PNH is an acquired hematopoietic stem cell disorder caused by somatic mutations in the phosphatidylinositol glycan class A (PIGA) gene, leading to a deficiency of glycosylphosphatidylinositol (GPI)-anchored proteins such as CD55 and CD59 [2]. These proteins normally protect blood cells from complement-mediated lysis. Their absence in PNH results in intravascular hemolysis, thrombosis, and, in some cases, bone marrow failure [3].

Thromboembolism is the leading cause of mortality in patients with PNH, accounting for up to 67% of deaths [6]. While thromboses classically occur in atypical venous locations-such as hepatic, mesenteric, dermal, and cerebral veins-arterial events are increasingly recognized, albeit less frequently reported. In one study, arterial thrombosis occurred in approximately 15% of PNH-associated thrombotic events [7]. Our case is unusual in that the patient presented with multiple recurrent arterial cerebrovascular events in the absence of a known prothrombotic state, ultimately leading to a delayed diagnosis of PNH.

This case illustrates several key diagnostic challenges. The patient initially presented with bilateral cerebral infarctions-an uncommon finding in young individuals without cardiovascular risk factors. A comprehensive cardiac and vascular workup, including TTE, TEE, and vascular imaging, failed to reveal a primary embolic source. The recurrence of stroke following a negative hypercoagulability panel led to further evaluation, during which laboratory signs of intravascular hemolysis were uncovered, prompting PNH testing. It is notable that normocytic anemia with elevated LDH and low haptoglobin levels was a subtle yet critical clue in this diagnostic process. Flow cytometry ultimately confirmed the diagnosis, revealing a large clone size (CD59-negative erythrocytes in 74%), which is strongly associated with an increased risk of thrombotic complications [8].

The mechanism of thrombosis in PNH is complex and multifactorial. Proposed contributors include complement-mediated platelet activation, nitric oxide depletion due to intravascular hemolysis, endothelial dysfunction, and increased levels of procoagulant microparticles [7]. Arterial thrombosis, while less commonly reported, may be underrecognized and can present with devastating complications such as myocardial infarction or ischemic stroke [8]. This case highlights the importance of considering PNH in the differential diagnosis of arterial thrombosis in young patients, particularly when standard workup is unrevealing and laboratory markers suggest hemolysis.

The advent of complement inhibitors, particularly eculizumab and its longer-acting counterpart ravulizumab, has transformed the prognosis of PNH. Eculizumab, a monoclonal antibody targeting complement protein C5, significantly reduces hemolysis, the incidence of thrombosis, and overall mortality in patients with classical PNH [10]. However, because eculizumab blocks the terminal complement cascade, it impairs the immune system’s ability to combat encapsulated organisms-most notably *Neisseria meningitidis*. As a result, patients must be vaccinated against meningococcal disease at least two weeks prior to initiating therapy when possible. If immediate treatment is necessary, prophylactic antibiotics are recommended until vaccine-induced immunity develops [11]. Despite vaccination, breakthrough infections have been reported; thus, patients and providers must remain vigilant for signs of meningococcal infection.

In our patient, the timely administration of the meningococcal vaccine and a two-week course of prophylactic antibiotics preceded eculizumab therapy. He tolerated treatment well and experienced no further thrombotic events in early follow-up.

This case underscores the critical need for a high index of suspicion for PNH in young patients with unexplained, recurrent thrombotic events-especially when accompanied by evidence of hemolysis. Early recognition and prompt initiation of complement blockade therapy are essential to reduce morbidity and prevent potentially fatal complications.

## Conclusions

This case highlights a rare and life-threatening presentation of PNH with recurrent arterial cerebral infarctions in a young, previously healthy patient. It underscores the importance of considering PNH in the differential diagnosis of unexplained thrombosis, particularly when accompanied by signs of hemolysis. Timely recognition and initiation of complement inhibition therapy can prevent further thrombotic complications and significantly improve clinical outcomes. Clinicians should maintain a high index of suspicion for PNH in atypical thrombotic presentations to ensure early diagnosis and management.

## References

[1] Jalbert JJ, Chaudhari U, Zhang H, Weyne J, Shammo JM (2019). Epidemiology of PNH and real-world treatment patterns following an incident PNH diagnosis in the US. Blood.

[2] Rollins SA, Sims PJ (1990). The complement-inhibitory activity of CD59 resides in its capacity to block incorporation of C9 into membrane C5b-9. J Immunol.

[3] Mastellos DC, Ricklin D, Yancopoulou D, Risitano A, Lambris JD (2014). Complement in paroxysmal nocturnal hemoglobinuria: exploiting our current knowledge to improve the treatment landscape. Expert Rev Hematol.

[4] Moyo VM, Mukhina GL, Garrett ES, Brodsky RA (2004). Natural history of paroxysmal nocturnal haemoglobinuria using modern diagnostic assays. Br J Haematol.

[5] Parker C, Omine M, Richards S (2005). Diagnosis and management of paroxysmal nocturnal hemoglobinuria. Blood.

[6] Hillmen P, Hall C, Marsh JC (2004). Effect of eculizumab on hemolysis and transfusion requirements in patients with paroxysmal nocturnal hemoglobinuria. N Engl J Med.

[7] Chatzileontiadou S, Hatjiharissi E, Angelopoulou M (2023). Thromboembolic events in patients with paroxysmal nocturnal hemoglobinuria (PNH): real world data of a Greek nationwide multicenter retrospective study. Front Oncol.

[8] Hill A, Kelly RJ, Hillmen P (2013). Thrombosis in paroxysmal nocturnal hemoglobinuria. Blood.

[9] Malato A, Saccullo G, Coco LL (2012). Thrombotic complications in paroxysmal nocturnal haemoglobinuria: a literature review. Blood Transfus.

[10] Kim JS, Jang JH, Jo DY (2023). Long-term efficacy and safety of eculizumab in patients with paroxysmal nocturnal hemoglobinuria and high disease burden: real-world data From Korea. J Korean Med Sci.

[11] Engel ER, Walter JE (2020). Rituximab and eculizumab when treating nonmalignant hematologic disorders: infection risk, immunization recommendations, and antimicrobial prophylaxis needs. Hematology Am Soc Hematol Educ Program.

